# Population pharmacokinetic analysis of sildenafil in term and preterm infants with pulmonary arterial hypertension

**DOI:** 10.1038/s41598-022-11038-6

**Published:** 2022-05-05

**Authors:** Su-jin Rhee, Seung Han Shin, Jaeseong Oh, Young Hwa Jung, Chang Won Choi, Han-Suk Kim, Kyung-Sang Yu

**Affiliations:** 1 Department of Pharmacy, Wonkwang University College of Pharmacy, Iksan, Republic of Korea; 2 Department of Pediatrics, Seoul National University Hospital, Seoul National University College of Medicine, Seoul, Republic of Korea; 3 Department of Clinical Pharmacology and Therapeutics, Seoul National University College of Medicine and Hospital, Seoul, Republic of Korea; 4 Department of Pediatrics, Seoul National University Bundang Hospital, Seoul National University College of Medicine, Seoul, Republic of Korea

**Keywords:** Paediatric research, Drug therapy

## Abstract

Sildenafil is widely used off-label in pediatric patients with pulmonary arterial hypertension (PAH). This study was conducted to characterize the pharmacokinetics (PK) of sildenafil in term and preterm neonates with PAH, by developing a population PK model, and to suggest appropriate doses to achieve clinically effective concentrations. A population PK modelling analysis was performed using sildenafil and its metabolite N-desmethyl sildenafil (DMS) concentration data from 19 neonates with PAH, whose gestational ages ranged 24–41 weeks. They received sildenafil orally at a dose of 0.5–0.75 mg/kg, four times a day. To investigate the appropriate sildenafil dose, simulations were conducted according to body weight which was significant covariate for sildenafil clearance. A one-compartment model with first-order absorption adequately described the PKs of sildenafil and DMS. Sildenafil clearance was expected to increase rapidly with increasing body weight. In the simulation, sildenafil doses > 1 mg/kg was required to achieve and maintain target concentrations of sildenafil and to expect timely clinical effects in term and preterm infants. These results could be utilized for the safer and more effective use of sildenafil in term and preterm infants.

## Introduction

Pulmonary arterial hypertension (PAH) is a life-threatening, progressive disease that is associated with high mortality and morbidity in children as well as adults^1^. In new-born infants, PAH is diagnosed by echocardiography based on findings such as tricuspid regurgitation, alignment of the intraventricular septum and the direction of shunt flow^2,3^. Various conditions can be attributed to the development of PAH in neonates. For term infants, the most common cause of PAH is persistent pulmonary hypertension in newborns (PPHN), and for preterm infants, bronchopulmonary dysplasia (BPD) can be accompanied by PAH in moderate to severe cases^4^. Currently, there are no established treatment guidelines for PAH in new-born infants. Inhaled nitrogen monoxide gas, sildenafil, endothelin receptor antagonist, and prostacyclin are used as clinical treatment drugs to help expand blood vessels, but no drugs have been approved for safety and effectiveness in newborns^5^.

Sildenafil is a selective and potent phosphodiesterase (PDE) type 5 inhibitor that interferes with the hydrolysis of cyclic nucleotides by PDE, through which cGMP accumulates, eventually releasing pulmonary blood vessels^6,7^. Since sildenafil was approved for the treatment of PAH in adults by the Food and Drug Administration (FDA) and the European Medicines Agency (EMA) in 2005, the drug has currently become the most widely used treatment for pediatric patients with PAH^5,6,8^. For pediatric patients with PAH, the dosage recommendation of sildenafil differs between the FDA and EMA. The FDA stated that the use of sildenafil is not recommended in children by revising drug labels in 2012^9^. However, the EMA allows the use of sildenafil for the treatment of pediatric patients aged 1–17 years, with 10 mg for patients with a body weight < 20 kg and 20 mg for patients with a body weight > 20 kg, three times a day^10^. Evidence of sildenafil therapy for neonates weighing less than 8 kg has not been established, and it is still used as an off-label drug, generally with a dose of 0.5–2 mg/kg^5,11^.

The off-label use of drugs may increase the risk of toxicity and other side effects, as well as uncertainty about their efficacy. In particular, in the case of newborns, there is a lack of clinical studies and information on appropriate treatment regimens, so it is necessary to generate a scientific basis for dosage regimens^12^. For sildenafil, several PK studies in neonates and premature infants have been reported. However, the evidence for the appropriate drug therapy of sildenafil in newborns, especially in premature infants, is still insufficient. Based on this understanding, the present study aimed (1) to quantitatively describe the pharmacokinetic (PK) properties of sildenafil in neonates, including premature infants with PAH, (2) to identify significant clinical factors affecting the PKs of sildenafil, and (3) to suggest an appropriate dose of sildenafil for neonates. To this end, a population PK modelling and simulation study was performed.

## Methods

### Patients and data collection

This study was an open-label, prospective study of oral sildenafil, which included term and preterm infants who received sildenafil for the treatment of PAH in the neonatal intensive care units at Seoul National University Hospital and Seoul National University Bundang Hospital. The patients were eligible to participate in the study if voluntary written consent was obtained from a parent, and patients were excluded if they had any of the following: a history of allergic reaction or known hypersensitivity to sildenafil; a hepatic impairment, which was determined as an aspartate aminotransferase (AST) level > 420 IU/L or an alanine aminotransaminase (ALT) level > 150 IU/L; had received vasopressors due to hypotension within the last 3 days; and had any clinical condition that researchers judged might make it difficult to conduct clinical trials. The study protocol was approved by the Institutional Review Board of Seoul National University Hospital and was carried out in concordance with the ICH Guidelines for Good Clinical Practice. Informed consent was obtained from parents for all participants. This study was registered at ClinicalTrials.gov as NCT02244528 (registered on 19/09/2014).

When neonate patients were diagnosed with PAH based on an echocardiogram, oral sildenafil dosing was started at 2 mg/kg/day 4 times a day (i.e., 0.5 mg/kg qid) for 2 weeks, based on their body weight. After 2 weeks, echocardiography was performed to evaluate the severity of PAH. If there was no clinical improvement or the right ventricular systolic pressure/systolic blood pressure did not decrease by more than 20% by echocardiographic findings, the sildenafil dose was increased to 3 mg/kg/day (0.75 mg/kg qid).

Blood sampling for PK analysis was performed up to 6 times per patient at the time of routine blood collections (e.g., for clinical laboratory tests) and was not performed for drug concentration measurements only. Sildenafil and DMS concentration measurements were performed after at least 4 drug administrations (i.e., 1 day after the start of drug administration). Even when the dose of sildenafil was changed, additional samples were collected after at least 4 doses of the altered sildenafil dose.

For the PK analysis of sildenafil and DMS, dosing information, including the dose, time of sildenafil administration, and blood sampling time, was collected. In addition, clinical and demographic characteristics, including sex, postnatal and gestational age, postmenstrual age (i.e., sum of the gestational age and postnatal age), current and birth body weight, indications, and laboratory test results (e.g., levels of AST, ALT, blood urea nitrogen [BUN], serum creatinine), were also collected and used for covariate assessment.

### Determination of sildenafil and DMS concentrations

Plasma concentrations of sildenafil and DMS, along with their respective internal standards, sildenafil-d8 and desmethyl sildenafil-d8, were quantified using liquid chromatography (Agilent 1260 series; Agilent Technologies, USA) and tandem mass spectrometry (API 4000; AB SCIEX, USA). Chromatographic separation was performed at 30℃ using a Luna C18 column (2.0 × 100 mm, 5 μm Phenomenex, USA) operated under isocratic conditions with mobile phase A (10 mmol/L ammonium acetate with 0.1% formic acid in distilled water) and mobile phase B (100% acetonitrile). The standard curve for sildenafil was linear in the range of 1–1,000 ng/mL, with a precision of ≤ 6.426% and an accuracy range of 96.02–102.8%, while the standard curve for DMS was linear in the range of 0.5–500 ng/mL, with a precision of ≤ 9.327% and an accuracy range of 95.60–102.1%.

### Population pharmacokinetic analysis

Population PK analysis of log-transformed plasma concentration–time data for sildenafil and DMS was performed using the nonlinear mixed effects modelling approach. The first-order conditional estimation with interaction method was implemented by NONMEM software (version 7.4, ICON Development Solutions, Ellicott City, MD, USA).

One- or two-compartment models with first-order absorption were evaluated to determine the best structural model to describe each of the concentration–time data of sildenafil and DMS. To analyse the sildenafil and DMS data simultaneously, the sildenafil dosage units were converted into micromoles, and sildenafil and DMS concentrations were converted to nanomolar concentrations based on molecular weights of 474.6 g/mol and 460.6 g/mol, respectively. Since N-demethylation of sildenafil to DMS by CYP3A4 (79%) and CYP2C9 (20%), which appears to be the only pathway of sildenafil biotransformation, is the main elimination pathway of sildenafil, it was assumed that sildenafil was completely metabolized to DMS in model development^13^.

Interindividual variability (IIV) of the PK parameters in the model was evaluated using an exponential error model, assuming that the IIVs follow a normal distribution with a mean of 0 and variance of ω^2^. Residual variability was tested using several residual error models, including additive, proportional, and combined additive and proportional error models. Finally, the residual variabilities were estimated using an additive error model separately for the sildenafil and DMS concentrations.

To explore the potential influence of covariates, demographic and clinical characteristic variables were tested graphically and statistically in the model. Continuous variables such as age (i.g., gestational age, postnatal age, and postmenstrual age), body weight, and laboratory test results, including levels of AST, ALT, BUN, and serum creatinine, were added into the model using the power functions (Eq. 1). In addition, age factors were also tested in the model using an exponential asymptotic model (Eq. 2), and categorical variables (i.e., sex) were added to the model using the following equation (Eq. 3):

1 $${TVP}_{j} = {\theta }_{n}\times {\left(\frac{{Var}_{j}}{Median\;or\;typical\;value}\right)}^{{\theta }_{m}}$$

2 $${TVP}_{j} = {\theta }_{n}\times \left(1-\mathrm{exp}\left(-\frac{\mathrm{ln}\left(2\right)}{{\theta }_{m}}\times {Age}_{j}\right)\right)$$

3 $${TVP}_{j} = {\theta }_{n}\times {{\theta }_{m}}^{{Var}_{j}}$$

where ${TVP}_{j}$ is the typical population estimate for the *j*th parameter, ${\theta }_{n}$ is the baseline population parameter estimate that is not explained by any of the included covariates, ${Var}_{j}$ represents the variable value in the *j*th patient; for sex, ${Var}_{j}$ is 1 if female, otherwise it is 0, and ${\theta }_{m}$ in Eq. (1) represents the exponent of the power function, while ${\theta }_{m}$ in Eq. (2) represents the maturation half-life of the age-related changes of the parameter, and ${\theta }_{m}$ in Eq. (3) represents the scaling factor for the covariate effect.

Model selection was performed in consideration of clinical relevance, based on the comparison of goodness-of-fit (GOF) plots and the likelihood ratio test. In particular, covariate assessment was performed using a stepwise forward selection and backward elimination approach. During forward selection, a decrease in the objective function value (OFV) greater than 3.84 (χ^2^, P ≤ 0.05 with 1 degree of freedom) between two nested models was considered significant. Then, an increase in the OFV of at least 6.63 (χ^2^, P ≤ 0.01 with 1 degree of freedom) was used as the criterion to retain the covariate in the model during the backward elimination procedure.

### Model evaluation

Throughout the model development process, the adequacy of the model was evaluated based on the GOF plots, the feasibility of the parameter estimates, and the precision of the parameters. In addition, the bootstrap resampling method was used to evaluate the stability of the final model. The predictive performance of the model was graphically evaluated by a prediction-corrected visual predictive check (pcVPC), which was performed each for sildenafil and DMS. Accordingly, the observed 5th, 50th, and 95th percentiles were plotted and compared against their respective simulated 90% confidence intervals.

### Simulation to investigate an optimal sildenafil dose

Based on the final model, a simulation was performed to predict the PKs of sildenafil and DMS and to determine the appropriate dose per body weight, which was identified as a significant covariate for sildenafil clearance. Virtual patients with body weights ranging from 0.8 to 4.2 kg were generated, and then the concentrations of sildenafil and DMS were simulated for the cases where 0.5–2 mg/kg (i.e., 0.5, 0.75, 1, 1.25 1.5, 1.75, and 2 mg/kg) of sildenafil was administered 4 times a day for 2 weeks.

The maximum concentration (C_max_) and the area under the concentration–time curve (AUC) after the repeated administration of sildenafil were estimated as indices of sildenafil exposure to investigate the appropriate dose of sildenafil based on the rate of reaching therapeutic exposure. The C_max_ and the AUC were calculated by applying non-compartmental analysis method to the simulated sildenafil and DMS concentration data. The therapeutic exposure references for the C_max_ and AUC were set based on the previously reported therapeutic exposure of sildenafil in infant patients as follows: therapeutic C_max_ references of sildenafil were 47, 140, and 373 ng/mL, which are expected to inhibit PDE 5 activity in vitro by approximately 53%, 77%, and 90%, respectively^14^; and the therapeutic exposure target for AUC, the combined AUC of sildenafil and DMS for 24 h, was set at > 2650 ng·h/mL, which has shown clinical efficacy in infant patients previously. The AUC was calculated by adding 50% of the DMS AUC to the sildenafil AUC, since the activity of DMS has been known to be 50% of that of sildenafil^15,16^.

## Results

### Study population characteristics

Study subjects were enrolled from February 2015 to July 2016. A total of 99 PK samples from 19 term and preterm infants (10 males and 9 females) were collected and analysed in this study. Of the total patients, 7 were diagnosed with PPHN, 4 were diagnosed with congenital heart disease, and 6 were diagnosed with BPD. Gestational ages ranged from 24 to 41 weeks, the range of postnatal age was 5–98 days, and the body weight range was 0.79–4.09 kg. The median postnatal age was higher in females than in males, while the overall range of postnatal age was similar. In the case of body weight at birth, the median value was slightly larger in males, but there was no significant difference in the range between males and females. Clinical laboratory test values such as AST, ALT, and BUN levels did not show significant differences according to sex. Four subjects received concomitant medications with CYP3A enzyme inducing action (i.e., bosentan and phenobarbital), and other three subjects used drugs with the potential to inhibit CYP enzyme (i.e., fluconazole and ranitidine) (Table 1). During the study period, the estimated right ventricular systolic blood pressure at baseline and after 2 and 5 weeks of sildenafil treatment were observed as follows: 50.9 mmHg at baseline (n = 15); 32.6 mmHg after 2 weeks (n = 14) and 24.0 mmHg after 5 weeks of treatment (n = 5).

**Table 1:** Clinical and demographic characteristics of the study population.

Characteristics	Male (n = 10)	Female (n = 9)	Total (n = 19)
Postnatal age (days)	7.5 (5–98)	42 (7–88)	11 (5–98)
Gestational age (weeks)	37 (27–41)	28 (24–41)	36 (24–41)
Body weight (kg)	3.21 (0.79–4.09)	3.11 (1.13–4.06)	3.18 (0.79–4.09)
Body weight at birth (g)	3095 (590–4270)	910 (690–3690)	2340 (590–4270)
AST level (IU/L)	32.5 (14–525)	30 (14–90)	30 (14–525)
ALT level (IU/L)	14 (5–117)	11 (6–34)	11 (5–117)
BUN level (mg/dL)	15 (5–22)	6 (4–26)	14 (4–26)
Serum creatinine level (mg/dL)	0.23 (0.09–0.86)	0.23 (0.20–0.46)	0.23 (0.09–0.86)
**Concomitant drugs affecting CYP enzymes**			
Bosentan	2 (20.0)	1 (11.1)	3 (15.8)
Fluconzaole	1 (10.0)	1 (11.1)	2 (10.5)
Phenobarbital	–	1 (10.0)	1 (5.3)
Ranitidine	1 (11.1)	–	1 (5.3)
**Indication**			
PPHN	4 (40.0)	3 (33.3)	7 (36.8)
CHD	2 (20.0)	2 (22.2)	4 (21.1)
BPD	2 (20.0)	4 (44.4)	6 (31.6)
Others	2 (20.0)	–	2 (10.5)

Data are presented as the median (range) except for concomitant drugs and indications, which are presented as the number of subjects (percentage, %). AST, aspartate aminotransferase; ALT, alanine aminotransaminase; BUN, blood urea nitrogen; CYP, cytochrome P450; PPHN, persistent pulmonary hypertension of the newborn; CHD, congenital heart disease; and BPD, bronchopulmonary dysplasia.

### Final Population Pharmacokinetic Model

**Figure 1:**
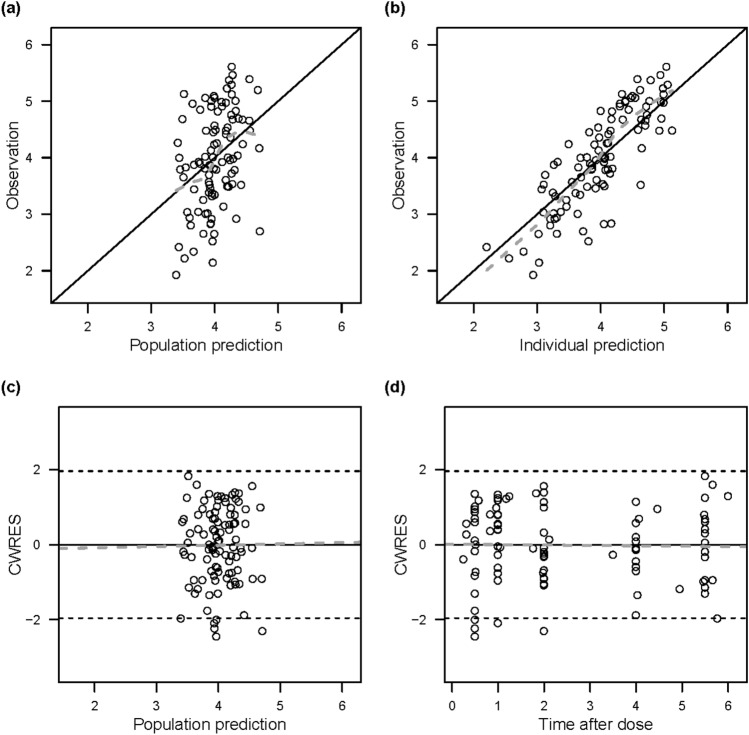
Goodness-of-fit plots for sildenafil. (**a**) Population predictions versus observed concentrations; (**b**) Individual predictions versus observed concentrations; (**c**) Conditional weighted residuals (CWRES) versus population predictions; and (**d**) CWRES versus time. The solid lines represent the line of unity. The dashed lines represent the linear regression fit.

**Figure 2:**
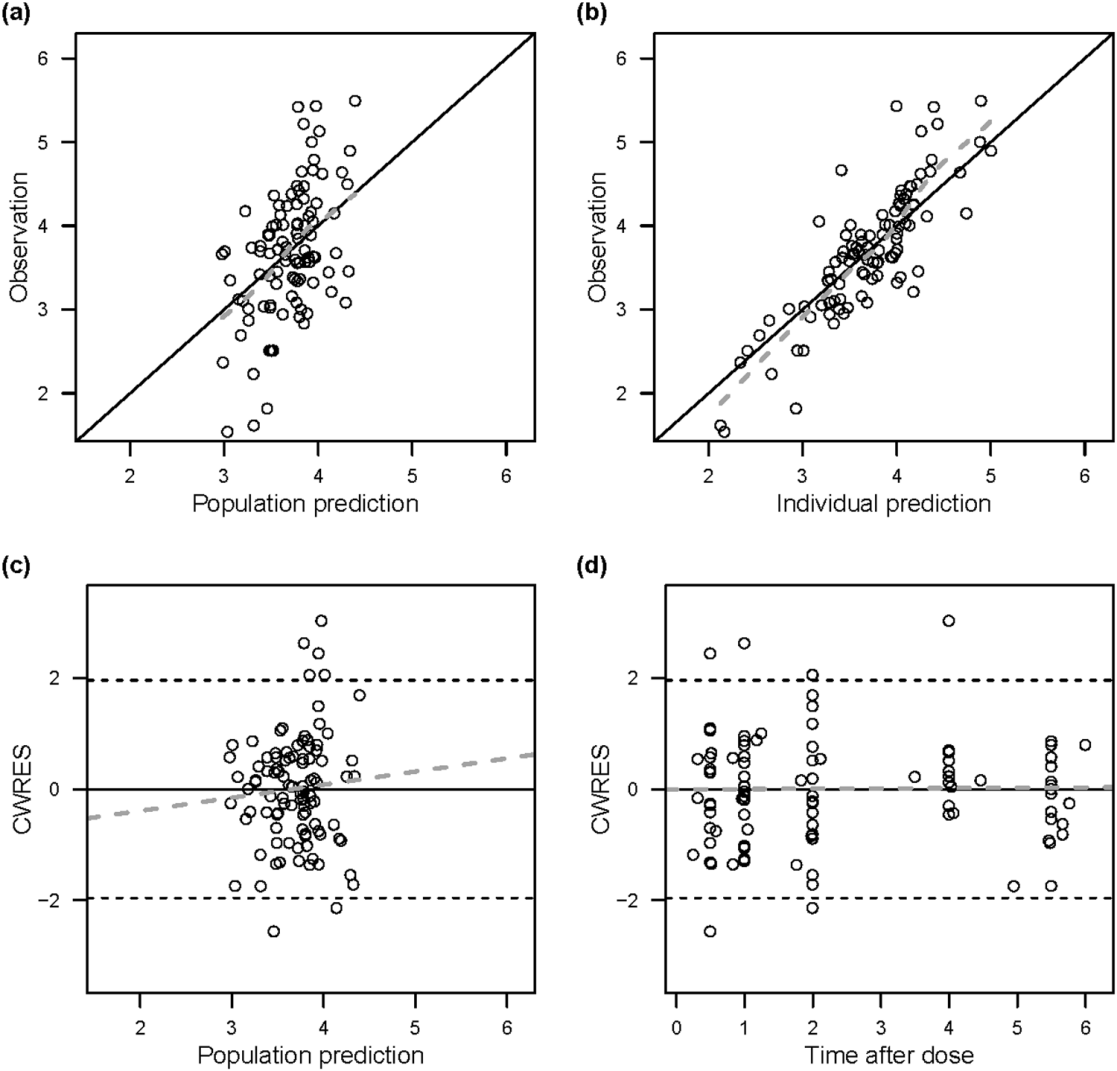
Goodness-of-fit plots for N-desmethyl sildenafil. (**a**) Population predictions versus observed concentrations; (**b**) Individual predictions versus observed concentrations; (**c**) Conditional weighted residuals (CWRES) versus population predictions; and (**d**) CWRES versus time. The solid lines represent the line of unity. The dashed lines represent the linear regression fit.

**Figure 3:**
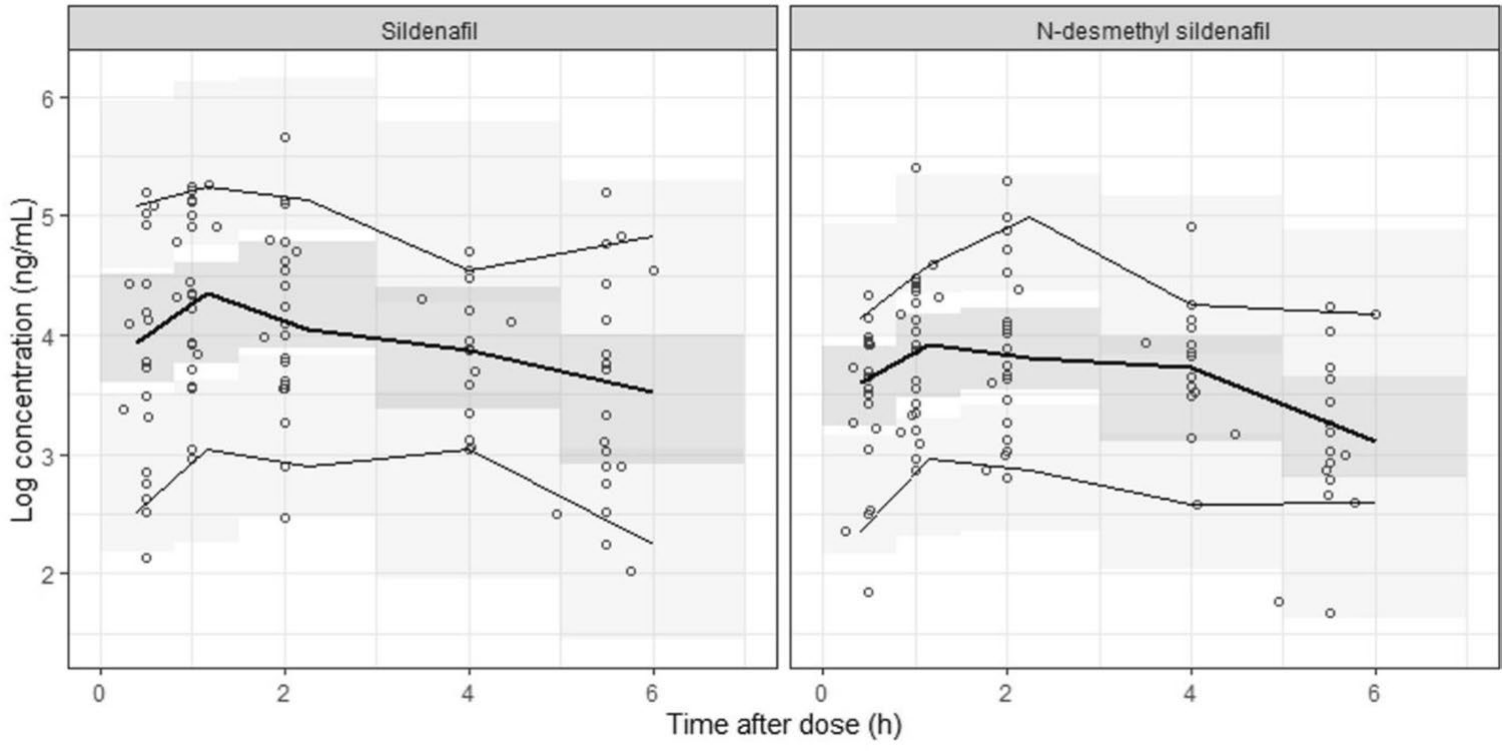
Prediction-corrected visual predictive check for sildenafil and N-desmethyl sildenafil. The solid lines represent the 5th, 50th, and 95th percentiles of the observed data. The shaded areas represent the 90% confidence interval of the 5th, 50th, and 95th percentiles of the predicted data.

The PKs of sildenafil and its metabolite, DMS, were each adequately described with a one-compartment model. The final model estimated the absorption rate constant of sildenafil, the apparent volume of distribution for sildenafil and DMS (i.e., V_sil_/F and V_DMS_/F’, respectively) and the apparent clearance for sildenafil and DMS (i.e., CL_sil_/F and CL_DMS_/F’, respectively). Since sildenafil was assumed to be completely metabolized to DMS and excreted, CL_sil_/F was estimated as the metabolic clearance (Table 2). For the final model, the GOF plots did not show any model misspecification: the population and individual predictions for sildenafil and DMS were evenly distributed around the line of identity with the observations, and the conditional weighted residuals were seen to be normally distributed (Figs. 1 and 2). The pcVPC plots for sildenafil and DMS showed a reasonable predictive ability of the final model: the 5th, 50th, and 95th percentiles of the observed data were mostly within the corresponding simulated data (Fig. 3).

**Table 2:** Parameter estimates of the final model.

Parameters	Estimates (RSE%)			Bootstrap median (95% CI)
	Base model	Full model	Final model	
Objective function value	35.689	0.031	15.055	
**Structural model**				
KA (h^−1^)	0.258 (20.1)	0.344 (22.1)	0.414 (10.7)	0.394 (0.216–0.810)
V_Sil_/F (L)	11.4 (38.6)	14.9 (31.0)	19.8 (31.4)	17.2 (7.57–41.2)
**CL**_**Sil**_**/F (L/h) = θ**_**CL(Sil)**_** × (body weight/3.14)**^**θweight**^** × θ**_**female**_** × (1−e**^**−θmaturation × postnatal age**^**)**				
θ_CL(Sil)_; typical value of CL_Sil_/F	9.27 (18.6)	8.87 (13.8)	10.1 (24.7)	10.1 (7.61–13.7)
θ_weight_; body weight exponent		1.06 (19.8)	0.899 (7.0)	0.848 (0.219–2.23)
θ_female_; sex effect		1.78 (16.1)		
θ_maturation_; postnatal age effect		4.28 (38.6)		
V_DMS_ /F’ (L)	2.51 (26.4)	2.26 (29.7)	1.78 (11.2)	2.11 (1.43–7.81)
**CL**_**DMS**_**/F’ (L/h) = θ**_**CL(DMS)**_** × (body weight/3.14)**^**θweight**^				
θ_CL(DMS)_; typical value of CL_DMS_/F’	12.4 (18.4)	14.4 (12.3)	14.3 (13.2)	14.2 (10.9–17.3)
θ_weight_; body weight exponent		1.26 (17.3)	1.34 (26.1)	1.32 (0.833–2.50)
**Interindividual variability (%CV)**				
V_Sil_/F	113.8 (37.7)	151.2 (22.2)	143.7 (19.0)	150.0 (61.6–315.2)
CL_Sil_/F	85.6 (23.4)	37.4 (17.6)	63.5 (17.7)	58.6 (29.4–86.3)
CL_DMS_/F’	88.5 (13.8)	49.4 (14.9)	49.2 (15.2)	47.6 (29.5–64.5)
**Correlation between etas**				
V_Sil_/F and CL_Sil_/F	0.261	0.249	0.295	0.245 (−0.068–0.629)
CL_Sil_/F and CL_DMS_/F’	0.453	0.104	0.178	0.166 (0.015–0.300)
V_Sil_/F and CL_DMS_/F’	0.175	0.0293	0.0606	0.061 (−0.274–0.341)
**Residual variability (SD)**				
Sildenafil	0.583 (9.1)	0.553 (8.0)	0.553 (7.7)	0.543 (0.443–0.626)
N-desmethyl sildenafil	0.487 (14.1)	0.474 (14.5)	0.472 (15.9)	0.451 (0.303–0.576)

KA, absorption rate constant; V_Sil_/F, apparent volume of distribution of sildenafil; CL_Sil_/F, apparent clearance of sildenafil; V_DMS_/F’, apparent volume of distribution of N-desmethyl sildenafil (DMS); CL_DMS_/F’, apparent clearance of DMS; CV, coefficient of variation; SD, standard deviation; RSE, relative standard error; CI, confidence interval.

In the final model, the IIV for V_sil_/F, CL_sil_/F and CL_DMS_/F’ were incorporated in the model. As a significant covariate of the PK parameter, the effects of body weight on CL_sil_/F and CL_DMS_/F’ were reflected in the model using a power function, since a significant positive correlation between body weight and those parameters were identified during the covariate assessment. Sex and postnatal age were also found to be significant covariates for CL_sil_/F in the full model, however, they were excluded in the final model: postnatal age was excluded as it did not meet the statistical significance criteria in the backward elimination process; on the other hand, sex was not reflected in the final model because there was no clear pharmacological explanation based on previous reports, although it was statistically significant (Table 2). Based on the final model, sildenafil elimination was expected to increase rapidly with increasing body weight.

### Optimal dose simulation

**Figure 4:**
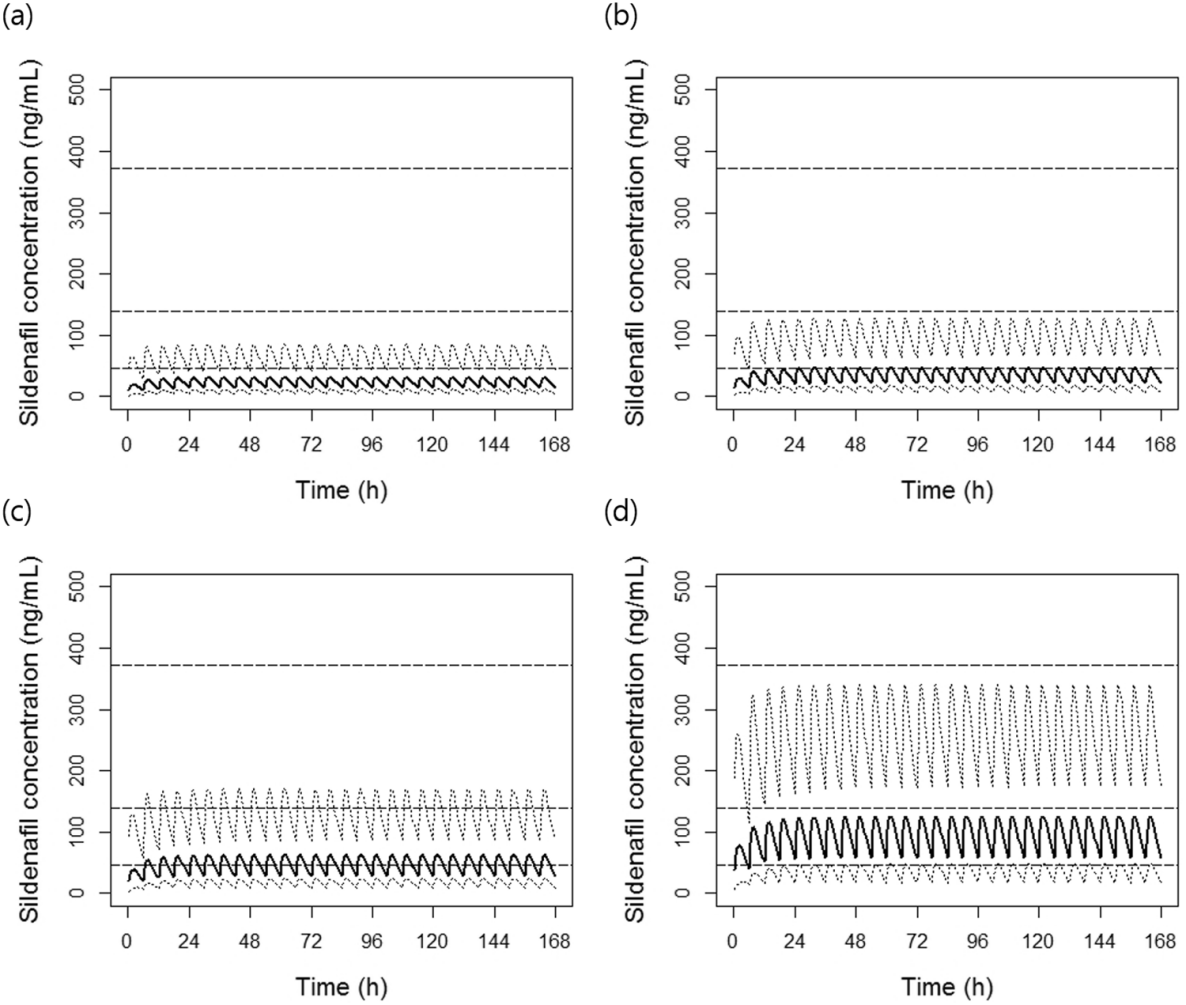
Simulated sildenafil concentration–time profiles after multiple administrations of sildenafil every 6 h: (**a**) 0.5 mg/kg; (**b**) 0.75 mg/kg; (**c**) 1 mg/kg; and (**d**) 2 mg/kg. The solid lines represent the 50^th^ percentiles, and the dotted lines represent the 5th and 95th percentiles of the simulated data. The dashed lines represent the therapeutic exposure references for C_max_: 47, 140, and 373 ng/mL are expected to inhibit type 5 phosphodiesterase activity in vitro by approximately 53%, 77%, and 90%, respectively^14^.

**Figure 5:**
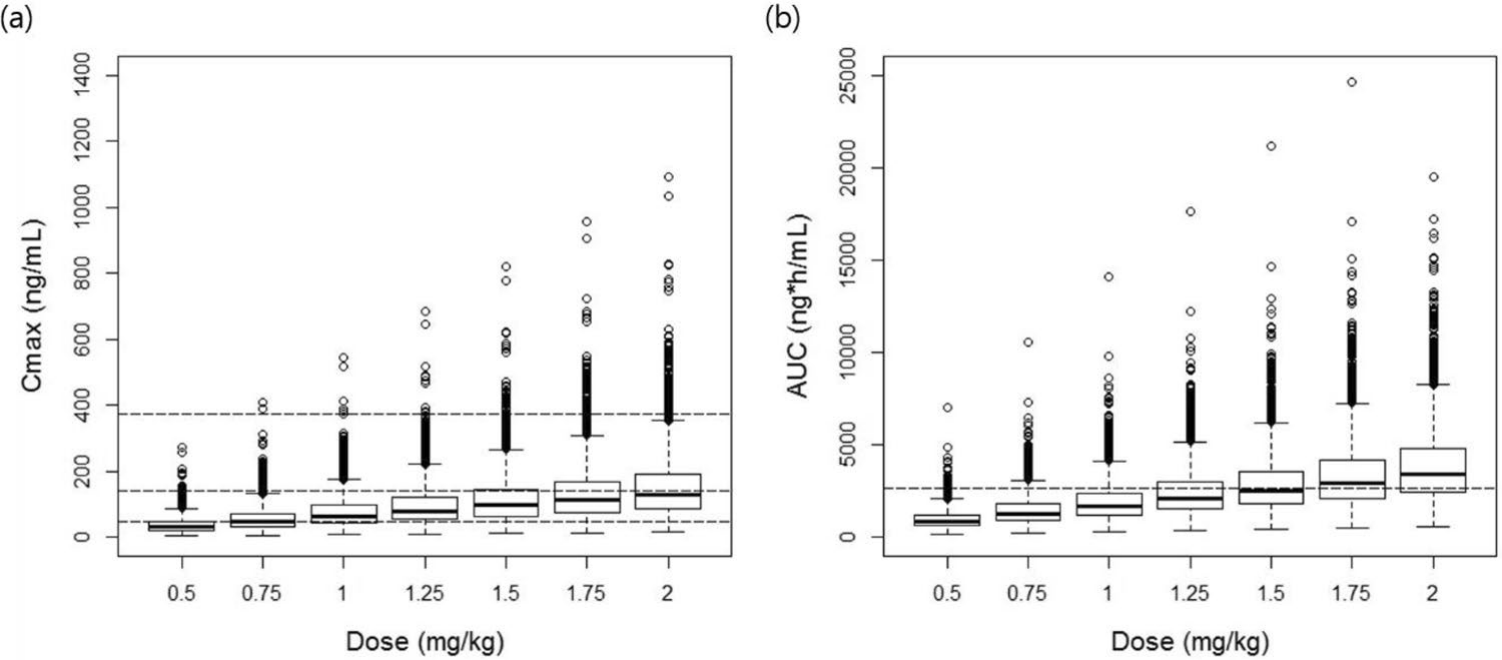
The predicted (**a**) C_max_ and (**b**) AUC at a steady state after multiple administrations of 0.5–2 mg/kg of sildenafil every 6 h. The dashed lines represent the therapeutic exposure references: the C_max_, 47, 140, and 373 ng/mL are expected to inhibit type 5 phosphodiesterase activity in vitro by approximately 53%, 77%, and 90%, respectively^14^; and the AUC, sildenafil and N-desmethyl sildenafil combined with an AUC > 2650 ng∙h/mL which previously showed clinical efficacy in infant patients^15^.

In the simulation using the final model, when 0.5 mg/kg of sildenafil was repeatedly administered at 6-h intervals for 7 days, it was expected that the sildenafil concentration would mostly remain below the therapeutic concentration range (Fig. 4). In the case of AUC, it was expected that the AUC reference level (i.e., > 2650 ng∙h/mL) would not be reached in most cases at doses lower than 1 mg/kg (Fig. 5). These results suggest that 0.5 mg/kg of sildenafil, which is usually used as an initial dose in newborns, may not be sufficient to show clinical effects.

**Figure 6:**
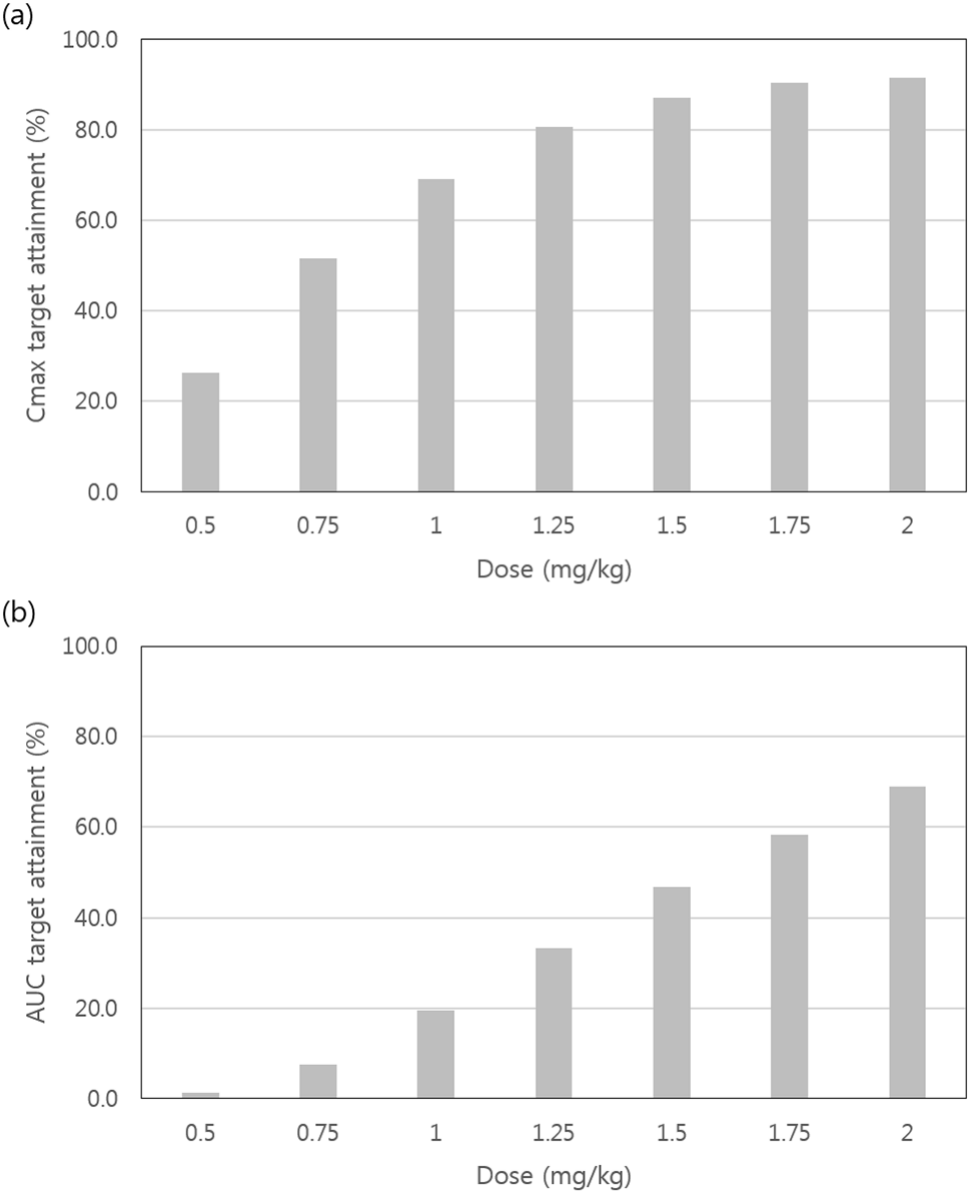
Percentage of reaching therapeutic exposure according to sildenafil dose. The therapeutic exposure for the (**a**) C_max_ and (**b**) AUC were set as follows: the C_max_, 47, 140, and 373 ng/mL, which are expected to inhibit type 5 phosphodiesterase activity in vitro by approximately 53%, 77%, and 90%, respectively^14^; and the AUC, sildenafil and N-desmethyl sildenafil combined with an AUC > 2650 ng∙h/mL, which previously showed clinical efficacy in infant patients^15^.

Accordingly, the percentages of reaching therapeutic exposure (i.e., a C_max_ of 47–373 ng/mL and an AUC > 2650 ng∙h/mL) were simulated to investigate the appropriate sildenafil dose for infant patients (Fig. 6). Based on the simulation results, when a sildenafil dose of 1 mg/kg was administered repeatedly 4 times a day, the rate of reaching the C_max_ target was expected to be approximately 70%. At the same time, it was expected that a dose of 1.25 mg/kg or higher would be required to reach the C_max_ target in more than 80% of the patients. On the other hand, the AUC target achievement rate was expected to be approximately 58.3% when a dose of 1.75 mg/kg was administered.

## Discussion

Sildenafil is a representative off-label drug used in neonates with PAH. Even if a drug is used off-label, the risk of side effects from drug use can be reduced if the scientific evidence for drug therapy is supported^17^. However, clinical trials are limited in children, especially in newborns, and it is practically difficult to generate scientific information on optimal drug therapy. Currently, for the treatment of PAH in neonates, sildenafil is used empirically without any guidelines for an appropriate dosing regimen. Several PK studies have suggested guiding information on sildenafil dosing in neonates, but have not provided confirmatory evidence; most of the studies have been conducted in a small number of subjects, whereas the PK variation of sildenafil in this neonatal population was relatively large^13,15,18–21^. Furthermore, to the best of our knowledge, few studies have been reported that characterized the PK of sildenafil in premature infants^19,20^. Therefore, it is very important to present new data and accumulate evidence for the treatment of sildenafil in premature infants. In that respect, the present study is clinically meaningful in that it generated evidence for the appropriate drug therapy of sildenafil in neonates, including premature infants. In this study, we successfully described sildenafil PKs quantitatively in neonates using sparse data by performing a population PK modelling analysis. In addition, we evaluated and suggested an appropriate sildenafil dose for extremely premature infant patients according to body weight, which is an important clinical factors.

In a previous study evaluating the PKs of sildenafil, it was suggested that a dose of 0.5–2 mg/kg of sildenafil 4 times daily in infants provides an exposure comparable to 20 mg 4 times daily in adults^15^. Accordingly, sildenafil, used off-label in neonates, is generally administered at a dose of 0.5–2 mg/kg 3 or 4 times a day^5,11^. Based on the simulated results in this study, however, it seems that sildenafil should be administered at a dose more than 1 mg/kg 4 times a day to achieve and maintain the target concentration of sildenafil and to expect a timely clinical effect. These results are consistent with the previously reported results of several population PK studies on infants that reported that when a dose of sildenafil less than 1 mg/kg is administered, it will be maintained at a low level compared to the therapeutic concentration range^15,19^.

Body weight-based dosing is commonly used in pediatric patients because it is easy to apply in clinical practice^22^. Sildenafil has also been used at a body weight-adjusted dose in children. In this study, the significance of various growth and development factors, including body weight, birth weight, gestational age, postnatal age, and postmenstrual age, were evaluated as covariates to explain IIV in the PKs of sildenafil in term and preterm newborn infants. As a result, body weight and postmenstrual age were identified as significant covariates. Since body weight and postmenstrual age are variables showing a strong correlation (Pearson's correlation coefficient was 0.803 with a P value < 0.001), only one of them (i.e., body weight) was reflected in the final model, based on the generality of clinical application, the degree of statistical significance, and the stability of the model parameter estimation. Accordingly, sildenafil doses for newborn infants were presented as doses per body weight. For the patients with a body weight range of 0.8–4.1 kg analysed in this study, the dose per body weight required to reach the therapeutic exposure was similar regardless of body weight, even when simulated by subdividing the body weight of each patient. This indicates that sildenafil can be administered to newborn infants at a dose per body weight to reduce IIV in its PKs according to growth and development. On the other hand, as previously reported, dose prediction for individual children may be adequately obtained only when the dose model is developed based on a narrow range of body weights or ages. That is, if a dose model is developed from neonates, then the dose should be predicted using the model only for neonates^23^. Accordingly, the dose suggested in this study should be applied only for term and preterm infants with a weight range of 0.8–4.1 kg.

During covariate evaluation in this study, sex was also identified as a significant covariate affecting the PKs of sildenafil in term and preterm new-born infants. That is, the clearance of sildenafil in females was estimated to be approximately twice that of males, so it was expected that the blood concentration of sildenafil would be lower in females, even at the same dose. This sex-specific clearance difference has not yet been reported in previous PK studies of sildenafil. One possible reason for the differences in clearance by sex might be that the postnatal age in females tended to be slightly higher than that in males, while the gestational age in females tended to be lower than in males in this study. However, the differences in postnatal age or gestational age according to sex was not statistically significant, and the effect of gestational age on sildenafil PKs was not significant in the covariate evaluation during model development. Furthermore, the significant effect of sex on sildenafil PKs was maintained even after postnatal age was reflected in the model, whereas the effect of postnatal age was not significant in the model reflecting sex. Consequently, it is necessary to pay attention to the possibility of differences in the PKs of sildenafil in preterm infants according to sex. Regarding CYP3A4, which contributes to approximately 80% of sildenafil metabolism, it has been reported that CYP3A4 expression is approximately twofold higher in women than in men in previous studies^24,25^. In addition, many drugs metabolized by CYP3A4 have been reported to exhibit higher clearance in women than in men, which persists even after correction for body weight^26–28^. In this regard, as the PK properties of sildenafil in premature infants have not been sufficiently studied, sex was excluded during model development in this study and further studies may be needed to clearly confirm the sex effect on the PKs of sildenafil in this population.

The therapeutic concentration range for sildenafil in children, particularly neonates, has not been clearly established. Accordingly, in most studies, the sildenafil dose in infants is estimated by targeting the AUC that showed a therapeutic effect in previous studies or the concentration level that showed inhibition of PDE in vitro as the therapeutic concentration range of sildenafil^15,19^. In this study, doses targeting both therapeutic C_max_ and AUC were also searched and presented. Although interindividual variation in sildenafil PKs has been accounted for through covariates such as weight, considerable variations still remained in the PK parameters (i.e., V_sil_/F, CL_sil_/F, and CL_DMS_/F’). In addition, especially in extremely young patients with incomplete maturation of metabolic enzymes, there would be large physiological changes according to growth and development, resulting in large IIV in the PKs of sildenafil^29–31^. Given that there is insufficient information on the efficacy and safety of sildenafil in neonates, it should be noted that dose adjustments of sildenafil should still be performed based on monitoring the clinical effects and adverse events.

There are several limitations in this study. First, the number of subjects included in the study was relatively small, and the sparse PK samples could not represent the entire PK profile of each individual subject. Consequently, it was necessary to use a simple one-compartmental model rather than a more sophisticated model to describe the PKs of sildenafil and DMS, which may not have precisely estimated the model parameters. Nevertheless, we successfully established a population PK model of sildenafil in neonates, and demonstrated the robustness and validity of the model. Second, this study could not find a significant effect of concomitant drugs on sildenafil PKs. As concomitant drugs varied according to each patient's condition, it was not possible to obtain statistical power to identify significant concomitant drugs through the covariate analysis. Considering previous reports of significant changes in sildenafil clearance by CYP3A inhibitors or inducers^15,19,32^, caution is warranted when coadministering sildenafil with drugs that affect CYP enzymes, especially 3A4.

In conclusion, this study established a population PK model that adequately describes the PKs of sildenafil in term and preterm infants with PAH, and presented the rationale for setting an appropriate body weight based dose. The results of this study are expected to be utilized as an evidence for more effective use of sildenafil, which is used off-label in newborn infants, for whom information on appropriate drug therapy is extremely limited.

## Data Availability

The datasets generated and/or analysed during the current study are available from the corresponding author upon reasonable request.

## References

[1] Tissot C, Beghetti M (2009). Advances in therapies for pediatric pulmonary arterial hypertension. Expert. Rev. Respir. Med..

[2] Bendapudi P, Rao GG, Greenougm A (2015). Diagnosis and management of persistent pulmonary hypertension of the newborn. Paediatr. Respir. Rev..

[3] Hoeper MM (2009). Definition, classification, and epidemiology of pulmonary arterial hypertension. Semin. Respir. Crit. Care Med..

[4] Steinhorn RH (2010). Neonatal pulmonary hypertension. Pediatr. Crit. Care Med..

[5] Dhariwal AK, Bavdekar SB (2015). Sildenafil in pediatric pulmonary arterial hypertension. J. Postgrad. Med..

[6] Singh TP (2010). Clinical use of sildenafil in pulmonary artery hypertension. Expert. Rev. Respir. Med..

[7] Leuchte HH, Schwaiblmair M, Baumgartner RA, Neurohr CF, Kolbe T, Behr J (2004). Hemodynamic response to sildenafil, nitric oxide, and iloprost in primary pulmonary hypertension. Chest.

[8] Ghofrani HA, Osterloh IH, Grimminger F (2006). Sildenafil: from angina to erectile dysfunction to pulmonary hypertension and beyond. Nat. Rev. Drug Discov..

[9] U.S. Food and Drug Administration. FDA Drug Safety Communication: FDA clarifies Warning about Pediatric Use of Revatio (sildenafil) for Pulmonary Arterial Hypertension. https://www.fda.gov/drugs/drug-safety-and-availability/fda-drug-safety-communication-fda-clarifies-warning-about-pediatric-use-revatio-sildenafil-pulmonary (2014)

[10] London, UK: European Medicines Agency. European Medicines Agency. Assessment report for Revatio. International non-proprietary name: Sildenafil. Procedure No. EMEA/H/C/000638/II/0028. https://www.ema.europa.eu/en/documents/variation-report/revatio-h-c-638-ii-0028-epar-assessment-report-variation_en.pdf (2011).

[11] Sola A, Baquero H (2007). Sildenafilo oral en medicina neonatal "Investigado para adultos, usado también por neonatos" [Oral sildenafil in neonatal medicine: "tested in adults also used in neonates"]. An Pediatr. (Barc)..

[12] Schrier L (2020). Off-label use of medicines in neonates, infants, children, and adolescents: A joint policy statement by the European Academy of Paediatrics and the European society for Developmental Perinatal and Pediatric Pharmacology. Eur. J. Pediatr..

[13] Mukherjee A, Dombi T, Wittke B, Lalonde R (2009). Population pharmacokinetics of sildenafil in term neonates: Evidence of rapid maturation of metabolic clearance in the early postnatal period. Clin Pharmacol Ther..

[14] Barst RJ (2012). A randomized, double-blind, placebo-controlled, dose-ranging study of oral sildenafil citrate in treatment-naive children with pulmonary arterial hypertension. Circulation.

[15] Ahsman MJ (2010). Sildenafil exposure in neonates with pulmonary hypertension after administration via a nasogastric tube. Arch. Dis. Child Fetal Neonatal. Ed..

[16] Mehrotra N, Gupta M, Kovar A, Meibohm B (2007). The role of pharmacokinetics and pharmacodynamics in phosphodiesterase-5 inhibitor therapy. Int. J. Impot. Res..

[17] Eguale T (2016). Association of off-label drug use and adverse drug events in an adult population. JAMA Intern Med..

[18] Huddleston AJ, Knoderer CA, Morris JL, Ebenroth ES (2009). Sildenafil for the treatment of pulmonary hypertension in pediatric patients. Pediatr. Cardiol..

[19] Gonzalez D (2019). Best Pharmaceuticals for Children Act - Pediatric Trials Network Steering Committee: Population pharmacokinetics of sildenafil in extremely premature infants. Br. J. Clin. Pharmacol..

[20] Thakkar N (2016). An opportunistic study evaluating pharmacokinetics of sildenafil for the treatment of pulmonary hypertension in infants. J. Perinatol..

[21] Cochius-den Otter SCM (2020). Pharmacokinetic modeling of intravenous sildenafil in newborns with congenital diaphragmatic hernia. Eur. J. Clin. Pharmacol..

[22] Hawcutt DB, Cooney L, Oni L, Pirmohamed M (2016). Precision dosing in children. Expert Rev. Precis. Med. Drug Dev..

[23] Mahmood I (2014). Dosing in children: A critical review of the pharmacokinetic allometric scaling and modelling approaches in paediatric drug development and clinical settings. Clin. Pharmacokinet..

[24] Wolbold R (2003). Sex is a major determinant of CYP3A4 expression in human liver. Hepatology.

[25] Waxman DJ, Holloway MG (2009). Sex differences in the expression of hepatic drug metabolizing enzymes. Mol. Pharmacol..

[26] Harris RZ, Benet LZ, Schwartz JB (1995). Gender effects in pharmacokinetics and pharmacodynamics. Drugs.

[27] Meibohm B, Beierle I, Derendorf H (2002). How important are gender differences in pharmacokinetics?. Clin. Pharmacokinet..

[28] Cummins CL, Wu CY, Benet LZ (2002). Sex-related differences in the clearance of cytochrome P450 3A4 substrates may be caused by P-glycoprotein. Clin. Pharmacol. Ther..

[29] Muirhead GJ, Wilner K, Colburn W, Haug-Pihale G, Rouviex B (2002). The effects of age and renal and hepatic impairment on the pharmacokinetics of sildenafil. Br. J. Clin. Pharmacol..

[30] Lacroix D, Sonnier M, Moncion A, Cheron G, Cresteil T (1997). Expression of CYP3A in the human liver–evidence that the shift between CYP3A7 and CYP3A4 occurs immediately after birth. Eur J Biochem..

[31] Koukouritaki SB (2004). Developmental expression of human hepatic CYP2C9 and CYP2C19. J. Pharmacol. Exp. Ther..

[32] U.S. Food and Drug Administration. REVATIO (sildenafil) Label. Reference ID: 3471998. https://www.accessdata.fda.gov/drugsatfda_docs/label/2012/021845s008lbl.pdf (2014).

